# Acute-on-chronic subdural hematoma: a new entity for prophylactic anti-epileptic treatment?

**DOI:** 10.1007/s00068-020-01508-9

**Published:** 2020-09-28

**Authors:** Sae-Yeon Won, Daniel Dubinski, Thomas Freiman, Volker Seifert, Florian Gessler, Adam Strzelczyk, Juergen Konczalla

**Affiliations:** 1 Department of Neurosurgery, University Hospital, Goethe University, Schleusenweg 2-16, 60528 Frankfurt, Germany; 2 Department of Neurosurgery, University Medicine of Rostock, Rostock, Germany; 3 Department of Neurology and Epilepsy Center Frankfurt Rhine-Main, University Hospital, Goethe-University, Frankfurt am Main, Germany

**Keywords:** Acute-on-chronic subdural hematoma, Seizure, Status epilepticus, Outcome

## Abstract

**Purpose:**

Acute-on-chronic subdural hematoma (acSDH) describes acute bleeding into a chronic subdural hematoma (SDH), after surgery or second trauma. Because seizures are a well-known complication of SDH, associated with substantial morbidity and mortality, we aimed to analyze the incidence of acute symptomatic seizures (ASz), including status epilepticus, and determine the functional outcomes in this specific cohort of patients.

**Methods:**

A retrospective analysis was performed, including patients with acSDH who were admitted to our department between 2010 and 2019. The incidence and timely onset of ASz and status epilepticus were evaluated. Functional outcomes at discharge and at 3–6 month follow-up were analyzed based on the modified Rankin scale.

**Results:**

Of 506 patients with chronic SDH, 29 patients (5.7%) were diagnosed with acSDH. The overall incidence of ASz and status epilepticus were 72.4% and 10.3%, respectively. Favorable outcomes were identified in 11 patients (52.4%) in the ASz group compared with 6 patients (75%) in the non-ASz group. The mortality rate was higher in the ASz group compared with that in the control group (29% vs 0%). At follow-up, favorable outcomes were similar to those observed at discharge (52.4% in the ASz group and 71.4% in the control group). The mortality rate was still higher in the ASz group, at 32% compared with 14% for the control group.

**Conclusion:**

AcSDH has a high risk for ASz, including status epilepticus, and is associated with unfavorable outcomes and high mortality. Thus, prophylactic treatment with antiepileptic drugs should be considered among this specific cohort of patients.

## Introduction

Chronic subdural hematoma (cSDH) is a disease with an increasing annual incidence in the United States, currently estimated at 7.2–10.3 per 100,000 persons and predicted to increase up to 17.1 per 100,000 persons by 2030, due to aging demographic alterations [13]. Elderly people are primarily affected by cSDH, which is associated with substantial morbidity and mortality. The relevance of cSDH has, therefore, increased in both clinical practice and research.

Seizures are well-known complications of cSDH, with an incidence rate ranging from 2 to 15.2%, and have been shown to be an independent predictor of unfavorable outcomes [24, 25]. Retrospective studies have investigated the effects of prophylactic treatment using antiepileptic drugs (AEDs); however, no general recommendation has been made, due to the low quality of evidence [18]. Some studies have reported reductions in seizure occurrence associated with the prophylactic administration of AEDs, whereas others have shown no beneficial effects associated with routine use [9, 13, 16, 19, 20]. These contrasting results regarding the prevention of seizures in cSDH have resulted in treatment discrepancies in clinical practice.

Acute-on-chronic SDH (acSDH) is defined by isolated acute intracranial bleeding into the subdural space, following burr hole trepanation of cSDH or trauma into the cSDH. The reported incidence of postoperative intracranial bleeding after the evacuation of cSDH is 4.6%, which often requires craniotomy resurgery procedures [17]. Acute bleeding into the subdural space has been reported as a significant predictor of seizures and status epilepticus. The irritation of the cortex by fresh erythrocytes and degradation products is believed to be the pathophysiological mechanism associated with the reduction of the epileptic threshold and seizure manifestation [5]. Thus, we hypothesized that acSDH patients who experience additional acute bleeding on the base of the cSDH may be at increased risk of seizure development and could represent a cohort among whom the routine use of prophylactic AEDs may be considered.

Here, our aim was to analyze the incidence of seizures and status epilepticus and the functional outcomes among patients with acSDH, to identify those who may benefit from prophylactic AED treatment.

## Methods

This study received internal review board approval from the local ethics committee. This study was performed as a retrospective study, including all patients with acSDH who were surgically treated at our department between January 2010 and December 2019. Patient consent was not deemed to be necessary for this study.

**Fig. 1:**
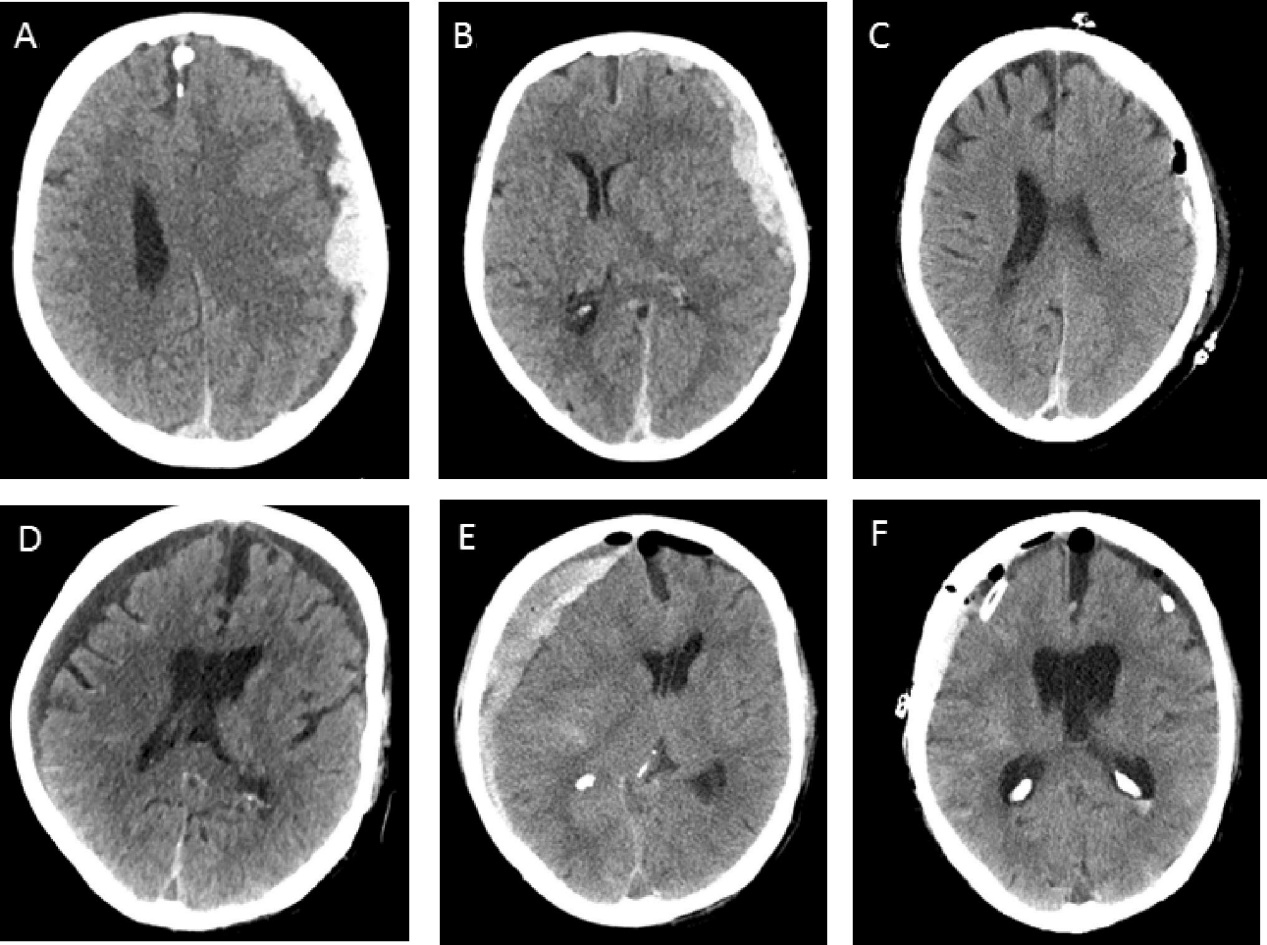
**a**, **b** Axial slice of a native CT-scan, showing acute bleeding into the previously detected cSDH, which was initially treated conservatively, due to anticoagulant intake and minimal clinical manifestation. **c** Postoperative axial slice of a native CT-scan, showing good postoperative SDH relief after burr hole evacuation. **d** Axial slice of a native CT-scan, showing a bilateral cSDH before bilateral burr hole evacuation. **e** Axial slice of a native CT-scan showing acute bleeding on the right side, postoperatively. **f** Axial slice of a native CT scan, after resurgery via craniotomy, on the right side. CT, computer tomography; cSDH, chronic subdural hematoma

The acSDH was defined as either isolated acute postoperative bleeding after the burr hole evacuation of cSDH or development of acute SDH, on the basis of cSDH, both requiring surgical treatment (Fig. 1). Patients with concomitant injuries like brain contusion or intracerebral hematoma diagnosed in a trauma CT scan were excluded from this study.

Data regarding demographic characteristics, symptoms at admission, pertinent medical history, use of anticoagulation or antiplatelet therapy, Glasgow coma scale (GCS) scores at admission and discharge, Glasgow outcome scale (GOS), and modified Rankin scale (mRS) scores at discharge and at 3–6-month follow-up were obtained from the prospective database. Favorable outcomes were defined as mRS scores between 0 and 3; otherwise, outcomes were defined as unfavorable.

Radiological information included acSDH volume and mid-shift, based on axial, non-contrast computed tomography CT imaging. Volumetric measurement was performed, as described previously [26]. Maximal deviation measurements of the midline structures, at the level of the foramen of Monroe, were used to calculate the midline shift.

Acute symptomatic seizures (ASz) were defined according to the International League Against Epilepsy (ILAE), based on clinical seizure manifestations, an ictal pattern in electroencephalogram (EEG) recordings, or clinical suspicion, with interictal epileptiform discharges in the EEG recordings and a temporal relationship with an acute brain insult [1]. Status epilepticus was defined as a generalized tonic–clonic seizure that lasts for more than 5 min or complex-partial seizures that last for more than 10 min, based on clinical observations or EEG recordings, for non-convulsive status epilepticus.

The primary study outcome was the incidence of ASz and status epilepticus in acSDH patients. The secondary outcomes included the functional outcomes at discharge and follow-up. Moreover, predictors for unfavorable outcomes were determined.

### Statistical analysis

Parameters, such as age, sex, symptoms at admission, comorbidities, and other quantitative parameters were analyzed. Hematoma volume and midline position are expressed as the median and interquartile range (IQR, 0.25–0.75). Comparisons among outcomes were performed using Fisher’s exact test and the Chi-squared test. For non-parametric statistical analysis, the Mann–Whitney *U* test was used. Statistical assessments were considered significant when *p* < 0.05.

## Results

### Patient demographics

**Fig. 2:**
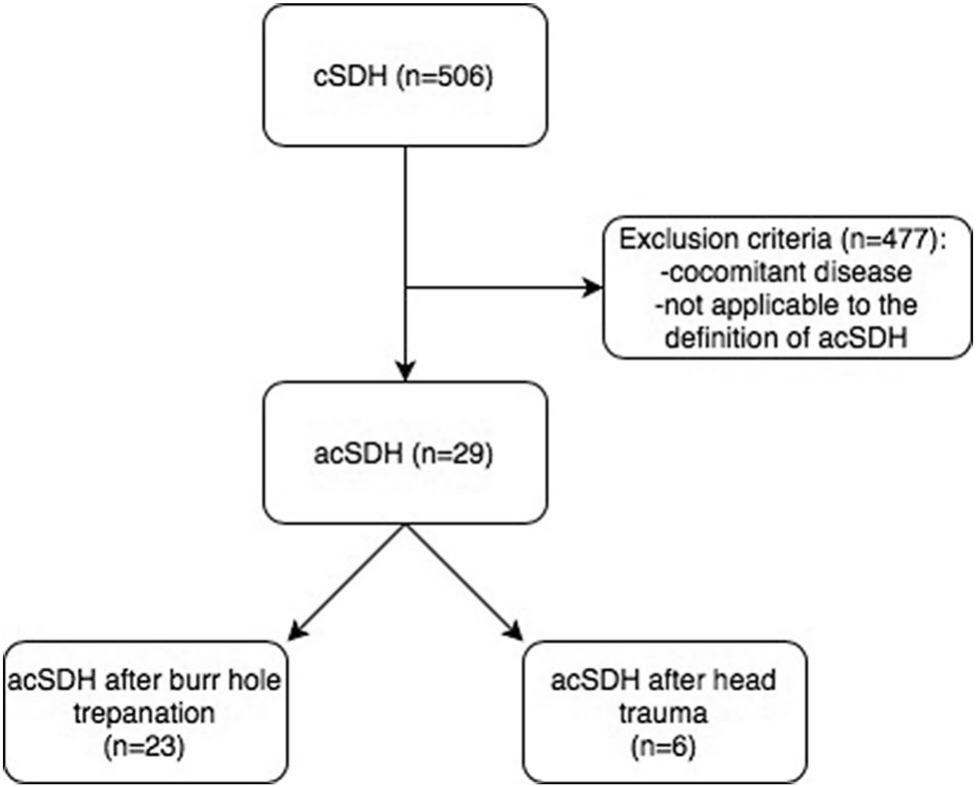
Incidence and timely onset of acute-symptomatic seizures and status epilepticus in acute-on-chronic SDH

In total, 506 patients with cSDH were entered into the prospective database during the 10-year study period. Among these, 29 patients (5.7%) were diagnosed with acSDH; 23 patients (4.5%) experienced acute rebleeding after a burr hole evacuation of a cSDH, and six patients (1.2%) experienced acute SDH within a preexisting cSDH due to head trauma by falling down on the ground (Fig. 2). The study population consisted of 13 females (44.8%) and 16 males (55.2%), with a median age of 79 years (IQR 70–86.1 years). At admission, 22 patients (75.9%) had GCS scores between 13 and 15, five patients (17.2%) had GCS scores between 9 and 12, and two patients (6.9%) had GCS scores between 3 and 8. The most common symptom at admission was gait impairment (51.7%), followed by paresis (37.9%), speech arrest (17.2%), impaired consciousness (17.2%), and seizures (10.3%). Seventeen patients (58.6%) were taking anticoagulant medications or antiplatelet agents compared to 240 of 506 patients with cSDH (47.4%) indicating a slight trend towards higher frequency, however, did not reach significance concerning rebleeding rate. The initial median hematoma volume was 146.3 cm^3^ (IQR 115.5–164.3 cm^3^), with a median midline shift of 6.5 mm (IQR 3.4–10.7). Detailed information can be found in Table 1.

**Table 1:** Basic characteristics

Number	29
Age, median [IQR], years	79 [70–86.1]
Sex (female)	13 (44.8%)
Medical history	
Hypertonus	20 (44.8%)
Diabetes mellitus type 2	10 (34.5%)
Atrial fibrillation	6 (20.7%)
Cardiovascular disease	7 (24.1%)
Coronary disease	10 (34.5%)
Respiratory disease	2 (6.9%)
Renal disease	9 (31.0%)
Metabolic disease	9 (31.0%)
Remote stroke/TIA	6 (20.7%)
Hematologic disease	5 (17.2%)
Oncology	4 (13.8%)
Drug history	
Anticoagulation	7 (24.1%)
-Marcumar	4 (13.8%)
-DOACs	3 (10.3%)
Antiplatelet	10 (34.5%)
GCS at admission	
GCS 13–15	22 (75.9%)
GCS 9–12	5 (17.2%)
GCS 3–8	2 (6.9%)
Symptoms at admission	
Impaired consiousness	5 (17.2%)
Vomiting	2 (6.9%)
Paresis	11 (37.9%)
Gait impairment	15 (51.7%)
Speech arrest	5 (17.2%)
Syncope	1 (3.4%)
Seizure	3 (10.3%)
Other symptoms	4 (13.8%)
CT	
Volume, median [IQR], cm^3^	146.3 [115.5–164.3]
Midline shift, median [IQR], mm	6.5 [3.4–10.7]
Side	
Left	10 (34.5%)
Right	8 (27.6%)
Both	11 (37.9%)

*IQR *interquartile range, *TIA *transient ischemic attack, *GCS *Glasgow coma scale

### Seizure and status epilepticus

**Fig. 3:**
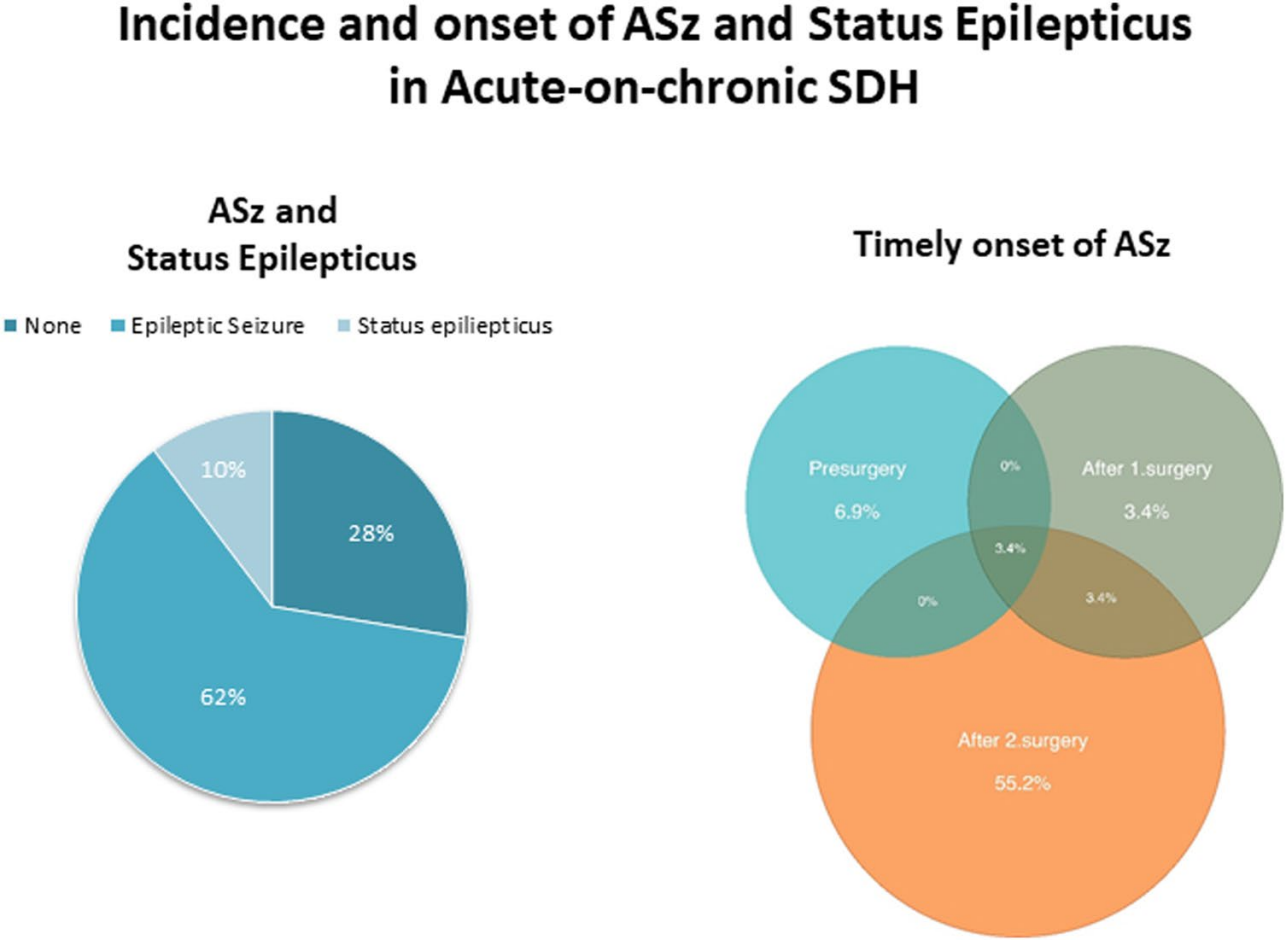
Modified Rankin Scale (mRS) scores for all patients, for patients with acute-symptomatic seizures, and for patients with no acute-symptomatic seizures, at discharge and follow-up

The overall incidence of ASz was 72.4%. ASz onset was most commonly observed following resurgery for the treatment of acSDH, in 18 patients (62.1%), among them 2 patients (6.9%) had additionally ASz prior to resurgery. The other 3 patients (10.3%) had ASz onset at admission. Status epilepticus was diagnosed in three patients (10.3%), one patient (3.4%) with focal and two patients (6.9%) with general status epilepticus (Fig. 3). No patients received prophylactic AEDs before the onset of seizures. Except for one patient (3.4%), all patients received levetiracetam as an anti-epileptic treatment, either as single or combined treatment (Table 2).

**Table 2:** Comprehensive data in patients with acute-on-chronic SDH

Case no.	Age (years), Sex	Diagnosis	Surgery	Complication	2. Surgery	Seizure	Pre-surgery	After 1. Surgery	After 2. Surgery	Status Epilepticus	Antiepilieptic treatment	mRS (discharge)	GOS (discharge)	mRS (follow-up)	GOS (follow-up)
1	44, M	cSDH	Burrhole	Acute-on-cSDH	Craniotomy	Yes	No	No	Yes	No	Valproat,Carbamazepin	0	5	0	5
2	72, M	cSDH	Burrhole	Acute-on-cSDH	Craniotomy	Yes	No	No	Yes	No	Levetiracteam, Lacosamid, Valproat,Topiramat	2	4	1	5
3	78, W	cSDH	Burrhole	Acute-on-cSDH	Craniotomy	No	No	No	No	No	No	3	3	1	5
4	79, M	cSDH	Burrhole	Acute-on-cSDH	Craniotomy	No	No	No	No	No	No	3	3	3	3
5	71, M	cSDH	Burrhole	Acute-on-cSDH	Craniotomy	Yes	Yes	No	No	No	No	3	4	2	4
6	86, W	cSDH	Burrhole	Acute-on-cSDH	Craniotomy	Yes	Yes	No	No	No	Levetiracetam	4	3	3	3
7	57, M	cSDH	Burrhole	Acute-on-cSDH	Craniotomy	No	No	No	No	No	No	3	3	2	3
8	82,W	cSDH	Burrhole	Acute-on-cSDH	Craniotomy	No	No	No	No	No	No	4	3	4	3
9	50, W	cSDH	Burrhole	Acute-on-cSDH	Craniotomy	No	No	No	No	No	No	3	3	2	4
10	70, W	cSDH	Burrhole	Acute-on-cSDH	Craniotomy	Yes	No	Yes	No	No	Levetiracetam,Valproat	4	3	3	3
11	96, M	cSDH	Burrhole	Acute-on-cSDH	Craniotomy	Yes	No	No	Yes	No	Levetiracetam	6	1	6	1
12	81. M	cSDH	Burrhole	Acute-on-cSDH	Craniotomy	Yes	Yes	Yes	Yes	No	Levetiracetam	5	2	NA	NA
13	70,W	cSDH	Burrhole	Acute-on-cSDH	Craniotomy	Yes	No	No	Yes	Yes	Levetiracetam, Lacosamid	5	2	5	2
14	91, M	cSDH	Burrhole	Acute-on-cSDH	Craniotomy	Yes	No	No	Yes	No	Levetiracetam	6	1	6	1
15	88, W	cSDH	Burrhole	Acute-on-cSDH	Craniotomy	Yes	No	No	Yes	Yes	Levetiracetam, Lacosamid	3	3	3	3
16	80, M	cSDH	Burrhole	Acute-on-cSDH	Craniotomy	Yes	No	No	Yes	No	Levetiracetam	2	4	2	5
17	75, M	cSDH	Burrhole	Acute-on-cSDH	Craniotomy	Yes	No	Yes	Yes	No	Levetiracetam,Lacosamid	4	3	5	3
18	90, M	Acute-on-cSDH	Craniotomy	No	No	No	No	No	No	No	No	3	3	NA	NA
19	88, W	cSDH	Burrhole	Acute-on-cSDH	Craniotomy	Yes	No	No	Yes	No	Levetiracetam	6	1	6	1
20	91, W	cSDH	Burrhole	Acute-on-cSDH	Craniotomy	Yes	No	No	Yes	No	Levetiracetam, Lacosamid	6	1	6	1
21	82, M	Acute-on-cSDH	Craniotomy	No	No	Yes	No	No	Yes	Yes	Levetiracetam	3	3	3	3
22	58, M	cSDH	Burrhole	Acute-on-cSDH	Craniotomy	No	No	No	No	No	No	1	5	0	5
23	80, M	cSDH	Burrhole	Acute-on-cSDH	Craniotomy	Yes	No	No	Yes	No	Levetiracetam	5	2	5	2
24	76, W	Acute-on-cSDH	Craniotomy	No	No	Yes	No	No	Yes	No	Levetiracetam	6	1	6	1
25	65, M	Acute-on-cSDH	Burrhole	No	No	Yes	No	No	Yes	No	Levetiracetam	1	5	1	5
26	80, M	Acute-on-cSDH	Craniotomy	No	No	Yes	No	No	Yes	No	Levetiracetam	2	4	NA	NA
27	72, W	cSDH	Burrhole	Acute-on-cSDH	Craniectomy	Yes	No	No	Yes	No	Levetiracetam	2	4	2	4
28	91, W	cSDH	Burrhole	Acute-on-cSDH	Craniectomy	Yes	No	No	Yes	No	Levetiracetam	6	1	6	1
29	69, W	Acute-on-cSDH	Craniotomy	No	No	No	No	No	No	No	No	5	2	6	1

### Surgical outcomes at discharge and follow-up

A total of 23 patients (79.1%) underwent initial burr hole trepanation for the evacuation of their cSDH, followed later by craniotomy due to rebleeding. In two of these patients, a third operation was necessary, consisting of a recraniotomy. The remaining six patients (20.9%) underwent an initial craniotomy and hematoma evacuation for acSDH. An overall favorable outcome was achieved in 15 patients (51.7%) at discharge. When the patients were divided into an ASz and a non-ASz group, favorable outcomes were achieved in 11 of 21 patients (52.4%) within the ASz group, compared with 6 of 8 patients (75%) in the control group. No patients from the non-ASz group died during the hospital stay, whereas six patients died from the ASz group, amounting to a mortality rate of 29%.

**Fig. 4:**
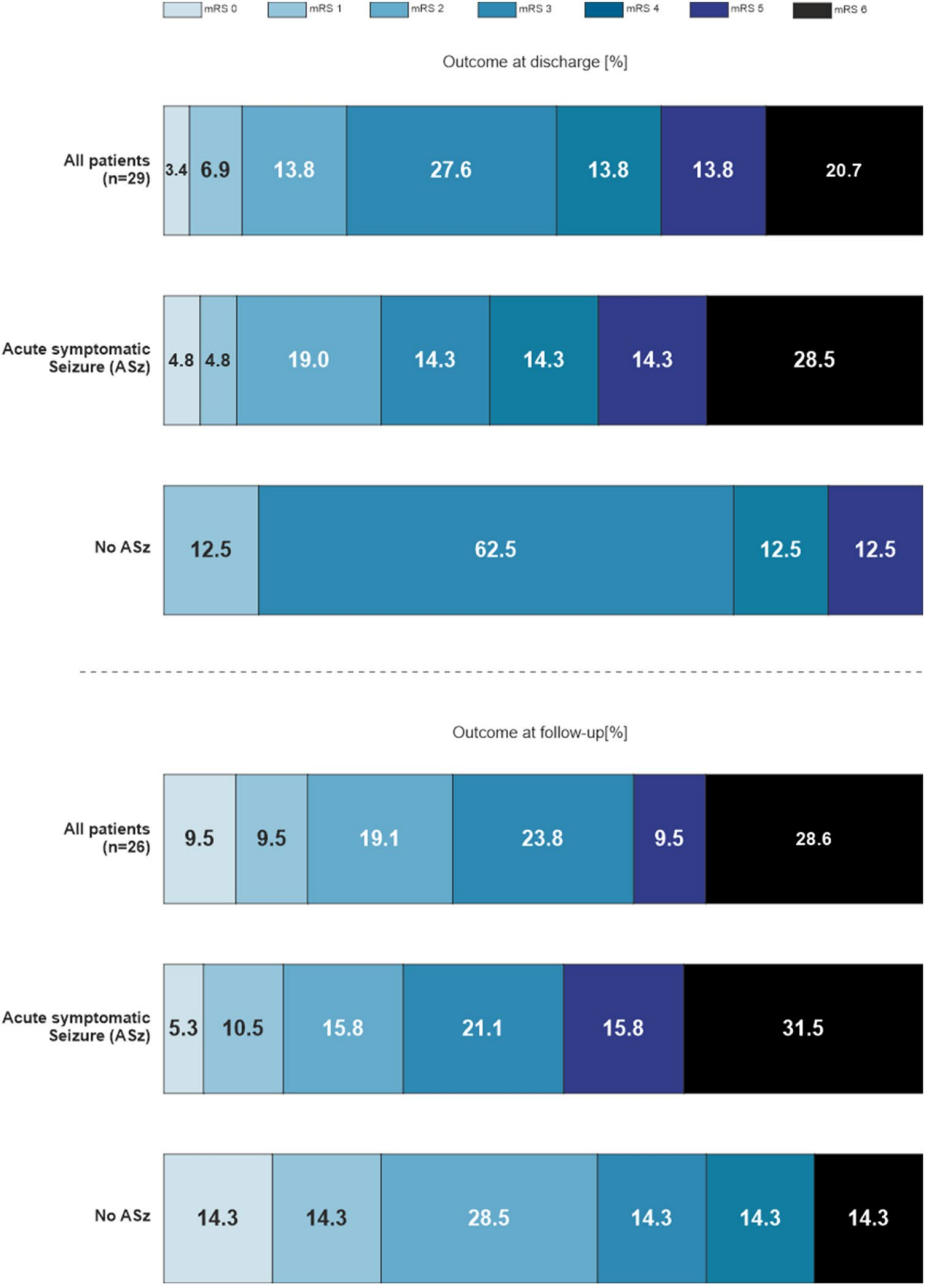
Functional outcome via modified Rankin scale in patients with acute symptomatic seizure and no seizure

At the 3–6-month follow-up time point, 26 patients (89.7%) were eligible for analysis. The rate of favorable outcomes at this time point was similar to those at discharge: 10 patients (52.6%) in the ASz group and 5 patients (71.4%) in the control group received favorable outcomes. Overall, a trend towards unfavorable outcomes and higher mortality rates was observed for the ASz group compared with the non-ASz group; however, these differences were not significant (Fig. 4).

The only identified predictor of unfavorable outcome at discharge was age > 80 years {*p* = 0.04; odds ratio (OR) 5.4; 95% confidence interval (CI) [1.0–27.6]} (Table 3). At follow-up, the number of cumulative comorbidities (*p* = 0.04, OR and 95% CI not available) and infections, including pneumonia and urinary tract infections (*p* = 0.01; OR 11.4′ Cl 95% [1.7–78.4] were predictors for unfavorable outcomes. Age > 80 years, the presence of cardiovascular disease, and GCS score < 13 showed trends towards predicting unfavorable outcomes at follow-up, but did not reach significance (Table 4).

**Table 3:** Predictors for unfavorable outcome at discharge

	Unfavorable outcome	Favorable outcome	*p *value
Number	14	15	
Age > 80 years	8 (57.1%)	3 (20%)	0.04
Medical history			
Hypertension	11 (78.6%)	9 (60%)	0.28
Diabetes mellitus	5 (35.7%)	5 (33.3%)	0.89
Atrial fibrillation	3 (21.4%)	3 (20%)	0.92
Cardiovascular disease	5 (35.7%)	2 (13.3%)	0.16
Coronary disease	4 (28.6%)	6 (40%)	0.52
Respiratory disease	2 (14.3%)	0 (0%)	0.13
Renal disease	6 (42.9%)	3 (20%)	0.18
Metabolic disease	4 (28.6%)	5 (33.3%)	0.78
Remote stroke/TIA	4 (28.6%)	2 (13.3%)	0.31
Hematologic disease	3 (21.4%)	2 (13.3%)	0.56
Oncology	3 (21.4%)	1 (6.7%)	0.25
Comorbidites, median [IQR]	4 [2.8–5.3]	3 [2–4.5]	0.17*
Anticoagulation/antiplatelet	9 (64.3%)	8 (53.3%)	0.55
GCS at admission			
3–8	2 (14.3%)	0 (0%)	0.13
9–12	3 (21.4%)	2 (13.3%)	0.56
13–15	9 (64.3%)	13 (86.7%)	0.16
Radiological parameter			
Unilateral	7 (50%)	11 (73.3%)	0.20
Midline shift, median [IQR]	6.5 [2.6–10.2]	6.5 [3.1–10.35]	0.53*
Volume, median [IQR]	146.2 [117.0–162.6]	148.6 [124.7–164.5]	0.75*
Infection	7 (50%)	4 (26.7%)	0.20
Pneumonia	4 (28.6%)	3 (20%)	0.59
Urinary tract	3 (21.4%)	1 (6.7%)	0.25
Seizure	12 (85.7%)	9 (60%)	0.12
Status epilepticus	1 (7.1%)	2 (13.3%)	0.58

*χ*^2^ test was used for parametric statistical analysis

*IQR *interquartile range, *TIA *transient ischemic attack, *GCS *Glasgow coma scale;

*Mann–Whitney *U*-test was used for non-parametric statistical analysis

**Table 4:** Predictors for unfavorable outcome at follow-up

	Unfavorable outcome	Favorable outcome	*p *value
Number	11	15	
Age > 80 years	6 (54.5%)	3 (20%)	0.07
Medical history			
Hypertension	9 (81.8%)	9 (60%)	0.23
Diabetes mellitus	5 (45.5%)	4 (26.7%)	0.87
Atrial fibrillation	3 (27.3%)	2 (13.3%)	0.37
Cardiovascular disease	4 (36.4%)	1 (6.7%)	0.06
Coronary disease	4 (36.4%)	4 (26.7%)	0.6
Respiratory disease	2 (18.2%)	0 (0%)	0.09
Renal disease	4 (36.4%)	3 (20%)	0.35
Metabolic disease	3 (27.3%)	3 (20%)	0.66
Remote stroke/TIA	2 (18.2%)	3 (20%)	0.91
Hematologic disease	3 (27.3%)	2 (13.3%)	0.37
Oncology	3 (27.3%)	1 (6.7%)	0.15
Comorbidities, median [IQR]	4 [2.3–5.8]	3 [2–4.5]	0.04*
Anticoagulation/antiplatelet	7 (63.6%)	7 (46.7%)	0.39
GCS at admission			
3–8	1 (9%)	0 (0%)	0.23
9–12	3 (27.3%)	1 (6.7%)	0.15
13–15	7 (63.6%)	14 (93.3%)	0.06
Radiological parameter			
Unilateral	6 (54.5%)	10 (66.7%)	0.53
Midline shift, median [IQR]			0.68*
Volume, median [IQR]			0.8*
Infection	7 (63.6%)	2 (13.3%)	0.01
Pneumonia	4 (36.4%)	2 (13.3%)	0.17
Urinary tract	3 (27.3%)	0 (0%)	0.03
Seizure	9 (81.8%)	10 (66.7%)	0.39
Status epilepticus	1 (9%)	2 (13.3%)	0.74

Chi-square test was used for parametric statistical analysis

*IQR *interquartile range, *TIA *transient ischemic attack, *GCS *Glasgow coma scale

*Mann–Whitney *U*-test was used for non-parametric statistical analysis

## Discussion

Our cohort study identified the high incidence of ASz (72.3%) and status epilepticus (10.3%) among patients with acSDH, which primarily occurred after the second surgical treatment. In particular, patients who experienced ASz were prone to experiencing unfavorable outcomes, with a mortality rate of approximately 30% at both discharge and at the 3–6-month follow-up time point. In addition, older age, infections, and the number of cumulative comorbidities were parameters associated with unfavorable outcomes.

Seizures and status epilepticus are conditions associated with reduced quality of life and increased mortality rates [10, 12, 15, 22]. Logroscino et al. followed patients for 10 years following their first status epilepticus episode and found that these patients died 2.8-fold more often than those in a matched-control group [15]. Moreover, individuals who experienced an acute symptomatic seizure were 8.9 times more likely to die within 30 years in a population-based study than those who did not, and several other studies have reported that seizure occurrence was associated with a significantly high rate of unfavorable outcomes [10]. [24] Consequently, some studies have examined the validity of prophylactic AED treatments. In cases of severe traumatic brain injury, level II evidence has been used to support the Brain Trauma Foundation guidelines that recommend the administration of prophylactic AEDs for one week [2]. However, no prospective, randomized studies have examined the use of prophylactic AED administration in patients with cSDH. Sabo et al. reported a significant reduction of seizures among patients treated with prophylactic AEDs, reducing from 32 to 2.4%, and Grobelny et al. identified AED prophylaxis as the only significant predictor of the lower incidence of postoperative seizures [9, 20]. However, other studies have reported a lack of beneficial effects following prophylactic AED administration, due to the incidence rate of seizures being too low to observe any effects [13, 16, 19]. We agree that a general recommendation for the use of AED prophylaxis is not justified in cSDH patients, without evidence in the literature; however, AED prophylaxis could be relevant for a select group of patients.

From a molecular and biological perspective, the pathophysiology of cSDH is based on neuroinflammation and angiogenesis, driven by vascular endothelial growth factor (VEGF), and clot formation, associated with increased bleeding and the hyper activation of fibrinolysis [3]. Cytokines, such as interleukin-1,-6, and-8, and matrix metalloproteinase also play roles in the development of the external and internal cSDH membrane. Angiopoietin-2 and VEGF also act as proangiogenic factors, sprouting leaky capillary vessels in the cSDH membranes [3]. In animal models, neuroinflammation, associated with the release of proinflammatory cytokines and other mediators, including cyclooxygenase and prostaglandin E synthase enzymes, has been shown to be involved in neurological disorders, including seizure and epilepsy [7]. Thus, neuroinflammation may be associated with the pathophysiological reduction in the epileptic threshold that facilitates seizure occurrence. Moreover, the hemolysis of fresh erythrocytes can cause the accumulation of reactive hemoglobin breakdown products and free iron, due to the breakdown of iron-rich compounds, such as hemosiderin [7]. In an experimental setting examining the iron-induced epilepsy model, the primary mechanisms associated with underlying seizures involved *N*-methyl-*D*-aspartate (NMDA)-receptor-mediated glutamate excitotoxicity, the development of reactive oxygen molecules, and subsequent membrane lipid peroxidation, resulting in neuronal cell death [7]. In view of these complex pathophysiological mechanisms, the high incidence of seizures and status epilepticus associated with acSDH is not surprising.

A recent study reported an incidence of ASz of 15.2% and an incidence of status epilepticus of 1.9% among cSDH patients, and seizures were associated with unfavorable outcomes. Several other studies have reported similar results regarding associations between seizures and high morbidity and mortality rates [6, 8, 11, 21]. In the current study, the incidence of ASz and status epilepticus in acSDH were approximately five times higher than those for the normal cSDH population; therefore, we believe that prophylactic treatment with AEDs should be considered for acSDH cases. Previous studies reported that the incidence of ASz is a risk factor for the development of structural epilepsy at the follow-up screening [4]. Because newer AEDs, such as levetiracetam and lacosamide, have been shown to be effective, with low complication rate and a very small interaction potential, prophylactic AED treatment for one week specific high-risk cohorts may represent an effective and well-tolerated seizure management strategy [14]. [23] However, further prospective trials remain necessary to validate the beneficial effects of prophylactic treatments with AEDs in acSDH patients.

## Limitations

By defining acSDH, we only included patients with acute bleeding in previously described cSDH. Consequently, some patients with acute SDH may have had a previously undetected or undiagnosed cSDH. Thus, selection bias may have occurred in this retrospective study. However a major strength is that none of our patients were treated with prophylactic AEDs; therefore, we present a homogenous cohort with notably high seizure risk, which may provide a sound base for designing a prospective study examining this specific cohort for further analysis, to further determine the usefulness of prophylactic AED treatment in this cohort.

## Conclusion

The incidence of ASz and status epilepticus in acSDH patients was notably high, occurring in 72.3% and 10.3% of cases, respectively, and associated with poor neurological outcomes and high mortality rates. In particular, these patients may benefit from prophylactic AED treatment; however, further studies are warranted prior to making any general recommendations.

## Data Availability

All data generated or analysed during this study are included in this published article.
